# Rational Design of Cellulose Nanofibrils Separator for Sodium-Ion Batteries

**DOI:** 10.3390/molecules26185539

**Published:** 2021-09-12

**Authors:** Hongyang Zhou, Jin Gu, Weiwei Zhang, Chuanshuang Hu, Xiuyi Lin

**Affiliations:** Key Laboratory for Biobased Materials and Energy of Ministry of Education, College of Materials and Energy, South China Agricultural University, 483 Wushan Road, Guangzhou 510642, China; zhouhy@stu.scau.edu.cn (H.Z.); gujin@scau.edu.cn (J.G.); zhangww@scau.edu.cn (W.Z.)

**Keywords:** cellulose nanofiber, sodium-ion batteries, separators, pore structure

## Abstract

Cellulose nanofibrils (CNF) with high thermal stability and excellent electrolyte wettability attracted tremendous attention as a promising separator for the emerging sodium-ion batteries. The pore structure of the separator plays a vital role in electrochemical performance. CNF separators are assembled using the bottom-up approach in this study, and the pore structure is carefully controlled through film-forming techniques. The acid-treated separators prepared from the solvent exchange and freeze-drying demonstrated an optimal pore structure with a high electrolyte uptake rate (978.8%) and Na^+^ transference number (0.88). Consequently, the obtained separator showed a reversible specific capacity of 320 mAh/g and enhanced cycling performance at high rates compared to the commercial glass fiber separator (290 mAh/g). The results highlight that CNF separators with an optimized pore structure are advisable for sodium-ion batteries.

## 1. Introduction

Cellulose is the most productive and widely distributed renewable resource in nature and can be derived from biomass [1,2,3]. Thanks to their multitude, durability and excellent mechanical properties, cellulose nanomaterials are increasingly used. A perfect example are cellulose nanofibrils (CNF), used as advanced functional materials in various fields, such as liquid crystal displays, coating various surfaces in the automotive industry and energy storage systems [4,5,6]. Owing to its advantages such as non-toxicity, excellent electrolyte compatibility and structural stability, CNF is considered to be a promising material to fabricate separators for metal-ion batteries. The application of CNF-based separators has been reported to demonstrate high safety and stable cycling performance in lithium-ion batteries (LIBs) [7,8,9].

With the increasing demand for LIBs, the scarcity and high cost of lithium resources have inevitably become potential problems. Therefore, sodium-ion batteries (SIBs) are developed as an alternative. SIBs have similar electrochemical mechanisms to LIBs [10]; at the same time, sodium resources are almost a thousand times more abundant than lithium on the earth [11]. However, because of the low solubility of sodium saults, high polarity solvents are used in the electrolyte for SIBs, requiring the separators to possess more affinity to electrolytes. Thus, the use of current commercial polyolefin separators in SIBs are restricted due to their poor electrolyte wettability. Glass fiber separator, although it shows excellent performance in SIBs, has a high price and poor mechanical properties which obstruct the large-scale use. Therefore, it is important to develop a low-cost and high-performance separator that can be matched with SIBs. Benefiting from the abundant functional groups in cellulose, CNF separators have excellent wettability to these polar electrolytes and attracted the researchers’ interests recently. Casas et al. prepared carboxymethyl cellulose (CMC) and hydroxyethyl cellulose (HEC) cross-linked separator, surpassing the performance of glass fiber and polyolefin separators in Na_3_V_2_ (PO_4_)_3_/Na half-cells [12]. Zhu et al., coated the ZrO₂ layer on the surface of the cellulose acetate membrane, in Na/hard carbon half-cell, a revisable capacity of ~280 mAh/g was achieved with the modified cellulose acetate membrane [13].

Nevertheless, few work reported the application of pure cellulose in SIBs up to date. Hence this study will explore the possibility of utilizing CNF separators for SIBs. Referring to the case in LIBs, CNF separators with higher porosity would demonstrate better electrochemistry performance [14]. Hence, it is hypothesized to obtain high-performance SIBs with porous CNF separator. Drying methods and solvents could effectively increase the porosity of the separator. Li et al. processed CNF foam by freeze-drying and monitored the freezing temperature and solvents to control its pore structure [15]. Chun et al. also modulated different porous structures by varying solvent mixture ratios in the CNFs suspension. The resultant separator exhibited highly interconnected nanoporous network channels and excellent mechanical properties, showed excellent rate capability and cycling performance in LIBs [16].

In this work, separators were fabricated using cellulose-based nanomaterials—CNFs by different methods to regulate the pore structure, including freeze-drying, air-drying, solvent-exchange, acid treatment and combination. The morphology and electrolyte uptake ability of CNF separator was studied. Then it was assembled into Na/hard carbon half-cell for testing the electrochemical performance. It was demonstrated that the CNF separators fabricated by freeze-drying and solvent exchange methods gave the highest porosity, and also possessed a high reversible capacity and good cyclability, outperforming the commonly used glass fiber separator in SIBs.

## 2. Results and Discussion

**Figure 1:**
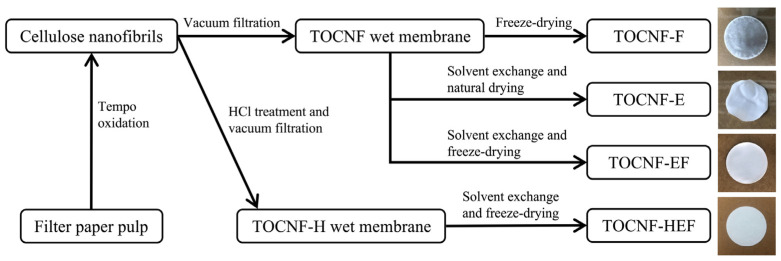
Flow chart illustrating the preparation process of TOCNF separators through freeze-drying, solvent exchange, freeze-drying after solvent exchanging, and acid-treated followed by freeze-drying after solvent exchanging.

The 2,2,6,6-tetramethylpyperidine-1-oxyl (TEMPO) oxidized CNF (TOCNF) separators were prepared using four methods (Figure 1), i.e., directly freeze-drying, naturally air-drying after solvent exchanging with ethanol and tert-butanol, freeze-drying after solvent exchanging, and acid-treated followed by freeze-drying after solvent exchanging; the obtained separator was named TOCNF-F, TOCNF-E, TOCNF-EF and TOCNF-HEF, respectively.

### 2.1. Morphology and Structure of TOCNF Separators

**Figure 2:**
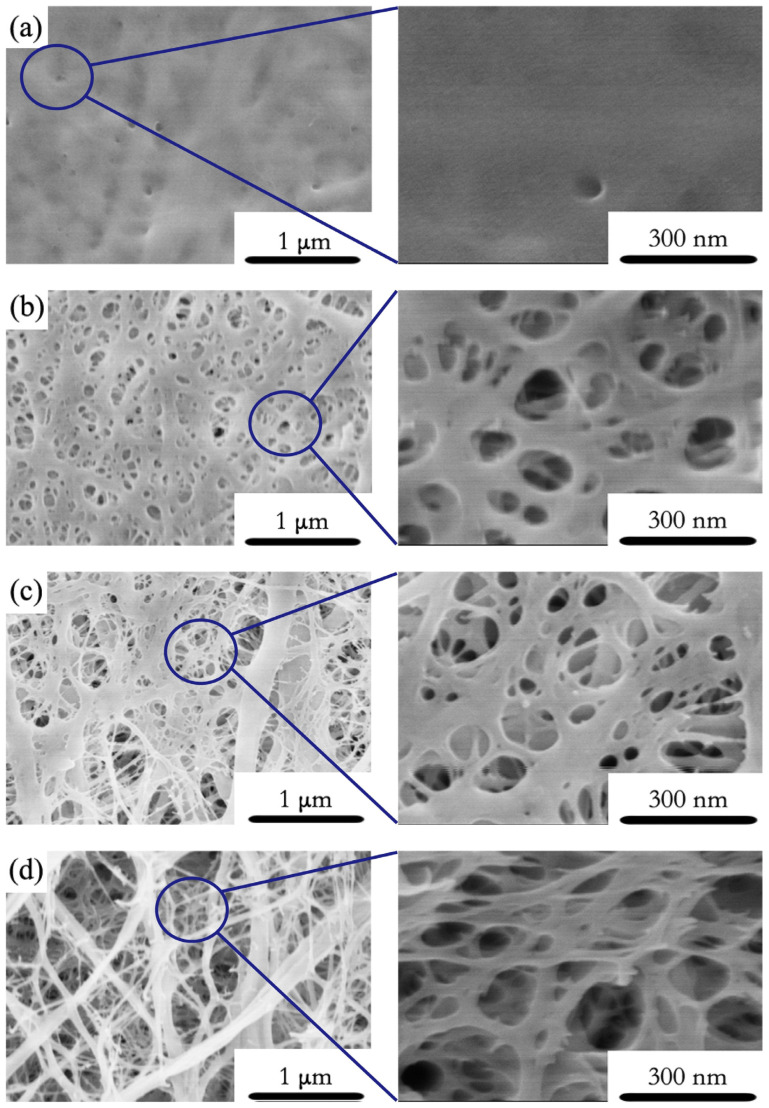
The SEM image of: (**a**) TOCNF-F; (**b**) TOCNF-E; (**c**) TOCNF-EF; (**d**) TOCNF-HEF at magnification of 30K (**left**) and 150K (**right**).

The SEM images of TOCNF-F, TOCNF-E, TOCNF-EF and TOCNF-HEF are shown in Figure 2. The carboxylated CNFs tended to aggregate by intermolecular hydrogen bonding and form a compact structure. Therefore, few pores were formed in TOCNF-F (Figure 2a). Through the solvent exchanging process, the intermolecular distance of CNFs was increased due to ethanol and tert-butanol, and uniform pores are generated in TOCNF-E during solvent evaporation, 100–300 nm in diameter (Figure 2b). Solvent exchange combined with freeze-drying resulted in the formation of the sparser pore structure, which can reach 100–1000 nm in size (Figure 2c,d). In addition, a large number of mesopores in tens of nanometers can be seen in TOCNF-E, TOCNF-EF and TOCNF-HEF according to SEM images at high magnification (150 K). Moreover, the HCl treatment increased protonation and lowered repulsion between CNFs, permitting larger pores in TOCNF-HEF. Jiang et al. [17] also found that highly porous structures are obtained from fully protonated CNFs where all surface carboxyl groups in the carboxylic acid form.

**Figure 3:**
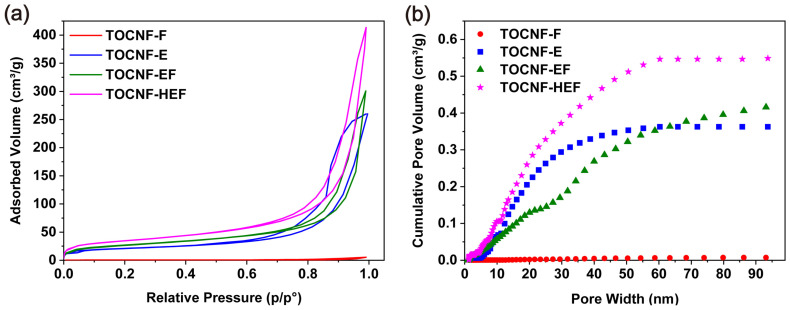
(**a**) Nitrogen adsorption–desorption isotherms and (**b**) cumulative pore volumes of TOCNF-F, TOCNF-E, TOCNF-EF and TOCNF-HEF separator.

To further identify the pore structure, the surface area was measured by the adsorption–desorption isotherm of N_2_. The BET surface area (Figure 3a) of TOCNF-F, TOCNF-E, TOCNF-EF and TOCNF-HEF is 1.9, 76.3, 95.2 and 123.5 m^2^/g, respectively. The BJH cumulative pore volume presents the same increasing trend, which is 0.0074, 0.3626, 0.4156 and 0.5485 cm^3^/g for TOCNF-F, TOCNF-E, TOCNF-EF and TOCNF-HEF, respectively (Figure 3b). Obviously, TOCNF-F displays an extremely low porosity that is unfavorable in enhancing the electrochemical performances of the cells. It can be seen that the solvent exchange greatly helps the formation of pores, and HCl treatment further increases the porosity. Both SEM and BET results revealed that TOCNF-HEF is more porous than other samples, and the sparser pore structures are effectively constructed after solvent exchange and freeze-drying.

**Figure 4:**
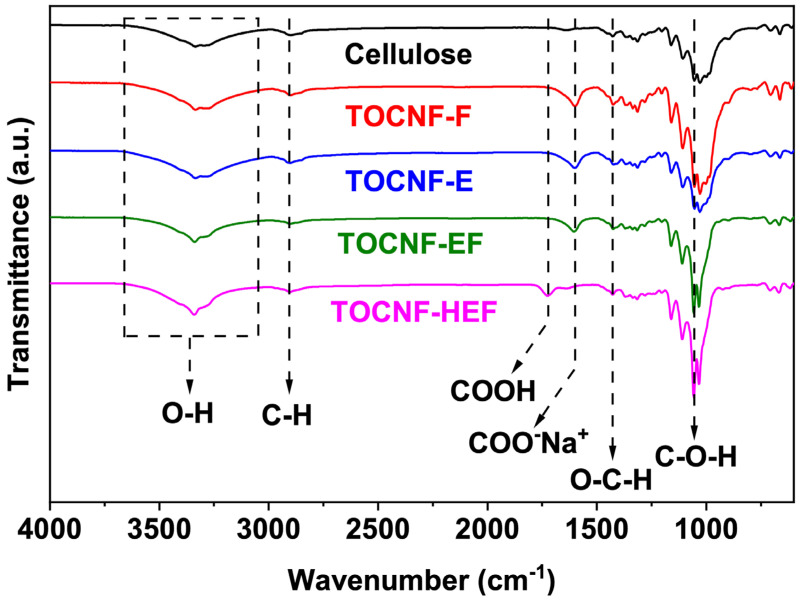
FTIR spectrum of original cellulose and TOCNF separators at range of 600–4000 cm^−1^.

The FTIR analysis was implemented to check the chemical groups on each separator, as shown in Figure 4. Original cellulose demonstrated absorption peaks at 3200–3700 cm^−1^ (O-H stretching), 2904 cm^−1^ (C-H stretching), 1421 cm^−1^ (O-C-H in-plane deformation) and 1042 cm^−1^ (C-O-H in-plane deformation). After TEMPO oxidation, a new peak at 1601 cm^−1^ appeared in TOCNF-F, TOCNF-E and TOCNF-EF, ascribed to carbonyl deformation of COO^−^Na^+^. In TOCNF-HEF, the characteristic peak of COO^−^Na^+^ disappeared, instead carbonyl stretching vibration at 1725 cm^−1^ was found, representing the complete conversion of COO^−^Na^+^ to COOH after acid treatment. These -COOH groups reduced the surface charge and permitted more substantial self-assembly [17], therefore TOCNF-HEF displayed larger pores in SEM.

### 2.2. Electrolyte Wettability of TOCNF Separator

The physical properties of separators are listed in Table 1. Since the four kinds of separators are assembled with the same number of CNFs, the thickness after different treatments can reflect the porosity of the separator to a certain extent. TOCNF-E (150 μm) was thicker than TOCNF-F (80 μm), indicating that solvent exchange through ethanol and tert-butanol can increase the intermolecular distance of CNFs to a greater extent. Moreover, the TOCNF-EF (240 μm) and TOCNF-HEF (250 μm) separator had larger thickness. The porosity of the TOCNF-F, TOCNF-E, TOCNF-EF and TOCNF-HEF separators was 37.1, 57.1, 70.8 and 74.6%, respectively. The higher porosity value of TOCNF-EF and TOCNF-HEF showed that combination of solvent exchange and freeze-drying could effectively expand the intermolecular distance between the CNFs. The electrolyte uptake of the TOCNF-F, TOCNF-E, TOCNF-EF and TOCNF-HEF separators was 206.2, 547.7, 776.7 and 978.8%, respectively. It is worth mentioning that the highest porosity and electrolyte uptake was obtained by TOCNF-HEF separator, together with a contact angle of 0°. This confirms the superior electrolyte wettability achieved by TOCNF-HEF, which is the fundamental requirement for high electrochemical performances.

### 2.3. Electrochemical Performances of TOCNF Separators

**Figure 5:**
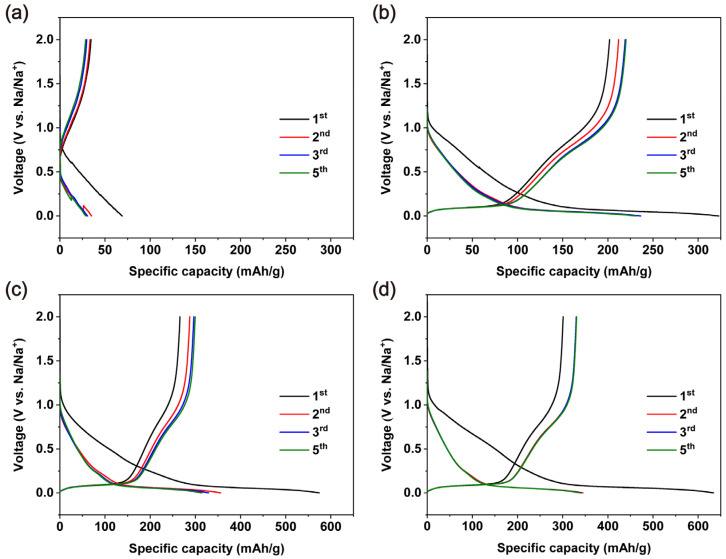
The voltage profile of 1st, 2nd, 3rd and 5th cycle at 25 mA/g at room temperature: (**a**) TOCNF-F; (**b**) TOCNF-E; (**c**) TOCNF-EF; (**d**) TOCNF-HEF.

Figure 5 showed the voltage profiles of Na/hard carbon cells using different separators. Due to the formation of a solid electrolyte interface (SEI) layer, the irreversible capacity loss was observed in the first charge/discharge cycle for all the cells [18]. The limited pores in the TOCNF-F separator resulted in poor performance, and the reversible specific capacity was only 35 mAh/g (Figure 5a). A reversible capacity of 220 mAh/g was achieved in TOCNF-E owing to the porous structure obtained from solvent exchange. The capacity further increased to 290 mAh/g when using the TOCNF-EF separator due to its highly porous structure, similar to the capacity reported using commercial glass fiber separators [19]. TOCNF-HEF achieved a highly reversible capacity of 320 mAh/g, and the charge/discharge curves of the second to fifth cycles were fully overlapped (Figure 5d). These results demonstrate the importance of the separator’s structure for high-performance SIBs. Interestingly, it was found that the capacity of the first cycle was lower than the following cycles in all the TOCNF separators; a possible explanation is that some blind holes gradually became permeable during the ion movement.

**Figure 6:**
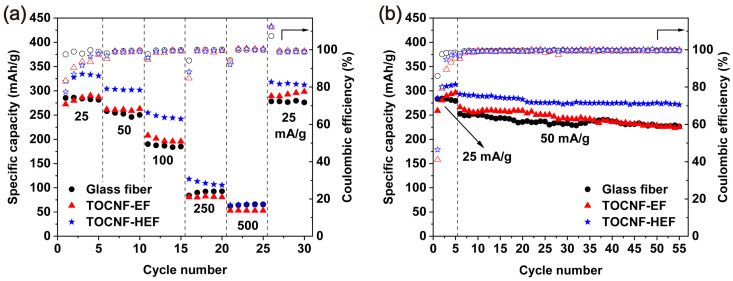
Electrochemical performance of TOCNF-EF and TOCNF-HEF at room temperature: (**a**) Rate performance at the rates of 25, 50, 100, 250 and 500 mA/g; (**b**) cycling performance at 25 mA/g for 5 cycles and then at 50 mA/g for 50 cycles under the voltage range of 0–2 V.

The TOCNF-EF and TOCNF-HEF are further compared with the glass fiber separator in terms of cycling performance. As shown in Figure 6a, TOCNF-EF had a charge capacity close to glass fiber, while TOCNF-HEF showed the highest capacity, particularly at the current density below 100 mA/g. At large current density, the performance of Na/hard carbon cell is mainly decided by the dynamic behavior of sodiation and desodiation in hard carbon [19], therefore the performance of all the separators is similar. When the current density returned to 25 mA/g, the capacity of all the three separators was recovered. Figure 6b revealed the cycling performance of the separators. The capacity retention rate after 50 cycles at 50 mA/g was 90.82, 87.01 and 93.80% for glass fiber, TOCNF-EF and TOCNF-HEF, respectively. TOCNF-HEF achieved the highest capacity and stability, thanks to a large number of uniformly distributed channels for Na^+^ transport, and polar chemical groups helped to form a stable interface between separator and electrode [20].

**Figure 7:**
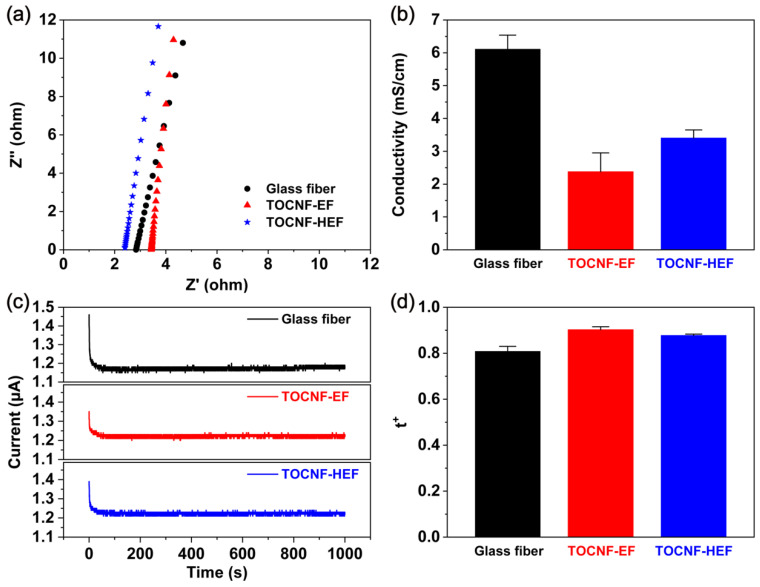
(**a**) The Nyquist plots of SS/separators/SS cells; (**b**) conductivity of glass fiber, TOCNF-EF and TOCNF-HEF; (**c**) amperometric i-t curves with a constant dc bias 10 mV; (**d**) Na^+^ transference number (t^+^) of glass fiber, TOCNF-EF and TOCNF-HEF. The above tests were conducted at room temperature.

The ionic conductivity and Na^+^ transference number were measured to further explain the excellent performance obtained by TOCNF-HEF. Figure 7a shows the Nyquist plots of the electrochemical impedance of symmetric cell using different separators. The high-frequency cutoff on the real axis reflects the impedance of Na^+^ transport in separators [21]. The lowest impedance of TOCNF-HEF is owing to its high porosity and great wettability to the electrolyte, which retains more electrolyte. As revealed in Figure 7b, the ionic conductivity of TOCNF-HEF (3.41 mS/cm) is higher than that of TOCNF-EF (2.38 mS/cm) separators, and glass fiber demonstrates an ionic conductivity as large as 6.11 mS/cm due to a rather huge porosity and large pores [22]. However, the electrochemical performances are mainly determined by the transport of Na^+^, hence the Na^+^ transference number of the separators were also analyzed.

Figure 7c shows the i-t curve of glass fiber, TOCNF-EF and TOCNF-HEF. When an over potential is applied, concentration gradients develop across the electrolyte because both cations (Na^+^) and anions (ClO_4_^−^) are mobile in the system. This would result in a time-dependent current until the concentration gradient reaches a steady state. The current decreases with the time and eventually stabilizes [23]. The Na^+^ transference numbers of glass fiber, TOCNF-EF and TOCNF-HEF were calculated to be 0.81, 0.90 and 0.88, respectively (Figure 7d). The TOCNF-HEF has a higher Na^+^ transference number than glass fiber does due to the presence of polar -COO^−^ groups, which inhibit the transport of ClO_4_^−^ in the electrolyte but are favorable for the passage of Na^+^ [24], and the repulsive force between CNFs makes it easy to form uniform and stable channels during the film formation process [14]. Hence, even though glass fiber displayed higher ionic conductivity, better electrochemical performances were obtained by TOCNF-HEF.

### 2.4. Mechanical Performances of TOCNF Separators

**Figure 8:**
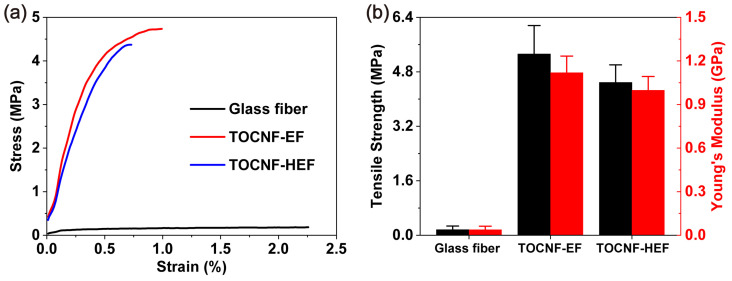
(**a**) Typical stress−strain curves of glass fiber and TOCNF separators; (**b**) tensile strength and Young’s modulus of the materials in (**a**). The above tests were conducted at room temperature.

Adequate mechanical strength can ensure the structural integrity of the separator, preventing the damage caused by the growth of dendrites and rough electrode surface, and thus is important to the safety of the battery. Figure 8 revealed the mechanical properties of glass fiber separators and TOCNF separators. The glass fiber separator has a tensile strength of only 0.16 MPa, whereas the tensile strength is 5.33 and 4.49 MPa for TOCNF-EF and TOCNF-HEF separators, respectively. In addition, the Young’s modulus of TOCNF-EF (1.12 GPa) and TOCNF-HEF (0.99 GPa) is significantly higher than that of glass fiber separator (0.04 GPa). The mechanical properties of TOCNF-HEF are slightly lower than those of TOCNF-EF, due to its higher porosity and more porous structure. The excellent mechanical property of TOCNF separators comes from two aspects. Firstly, cellulose nanofibrils are nanomaterials with an extremely high tensile strength and Young’s modulus [8]. Secondly, the interconnected porous structure of the separator maximizes the fiber reinforcement effect. The above results indicate that TOCNF-EF and TOCNF-HEF separators are much tougher and stronger than glass fiber separators and can ensure the safety of batteries.

### 2.5. Comparison with Other Separators for SIB

The main characteristics of CNF separators are compared with other separators reported in the literature in Table 2. Among the listed parameters, ionic conductivity, Na^+^ transference number and electrolyte uptake are the main concerns of researchers, which directly determined the electrochemical properties of the separator, while tensile strength reflects the safety and stability. Glass fiber and porous ceramic separator show high ionic conductivity and Na^+^ transference number, whereas its low tensile strength might bring some safety problems. On the contrary, polymer separators, including the commercial Celgard 2730 and polyvinylidene fluoride (PVDF) separators, demonstrate good mechanical properties; nevertheless, the ionic conductivity and Na^+^ transference number are low owing to the poor affinity to the electrolyte. The cellulose-based separators (TOCNF-HEF and CMC/HEC) exhibit both high ionic conductivity and acceptable mechanical property, particularly for the TOCNF-HEF separator prepared in this study presenting a high electrolyte uptake of 978.8% due the optimized pore structure. This attractive electrolyte wettability and absorption give a high ionic conductivity [25]. Adequate properties can be obtained by composite separators, such as the ZrO_2_-reinforced cellulose acetate membranes (ZrO_2_@MCA), agarose-based membranes with poly (vinyl alcohol) (Agarose/PVA) and cellulose–polyacrylonitrile–alumina composite (Cellulose-PAN-Al_2_O_3_). This is ascribed to the combine the advantages of several materials, thus remarkable electrochemical performance and mechanical are obtained simultaneously. Therefore, it is planned to hybrid TOCNF separators with other materials to further enhance the performance.

## 3. Experimental Section

### 3.1. Materials

Qualitative filter paper (medium speed, Whatman, Maidstone, England), hydrochloric acid (HCl, 36–38%), sodium hydroxide (NaOH, 96% Fuchen Chemical Reagent Co., Ltd., Tianjin, China), sodium hypochlorite (NaClO, 7.5%, Guangzhou Chemical Reagent Factory, Guangzhou, China), 2,2,6,6-tetramethylpyperidine-1-oxyl (TEMPO, 98%, Aladdin, Shanghai, China) and sodium bromide (NaBr, 99.0%, Fuchen Chemical Reagent Co., Ltd., Tianjin, China), ethanol absolute (C_2_H_6_O, 99.7%, General-reagent), tert-butanol (C_4_H_10_O, 99.0%, Maclean, Shanghai, China), hard carbon (Carbotron P, Kureha, Shanghai, China) and glass fiber separators (GF/D, Whatman, Maidstone, England) were used as received.

### 3.2. Preparation of Cellulose Nanofibrils

The qualitative filter paper was defibrillated into cellulose nanofibrils by TEMPO mediated oxidation by using a previously reported procedure [30]. Typically, 1 g qualitative filter paper was added into 100 mL DI water with 0.016 g TEMPO and 0.1 g NaBr, then mixed with 5 mmol NaClO to initiate oxidation reaction and conducted at pH of 9.8−10.2, adjusted by adding 0.5 M NaOH at room temperature. When the pH is constant, the oxidation reaction is considered complete. The obtained solution was centrifuged at 5000 rpm for 15 min, and the precipitate was collected, then was dialyzed against water by a dialysis separator (MWCO: 14,000 Da) to remove salts and other small molecules. The received product was firstly mechanically blended at 37,000 rpm for 30 min and then centrifuged at 5000 rpm for 15 min; the supernatant was collected and labeled as TOCNF.

### 3.3. Preparation of the TOCNF Separator

The TOCNF suspension was diluted to 0.1 wt% and vacuum filtered with a basis weight of 38 g/m^2^ to obtain wet separator. The wet separator was dried to prepare TOCNF separators. The acid-treated TOCNF separator (TOCNF-HEF) was prepared by adding 10 mM HCl to TOCNF suspension followed by solvent exchanging and freeze-drying [31]. 

### 3.4. Characterization of TOCNF Separator

Fourier transform infrared spectroscopy (FTIR, VERTEX 70, Bruker, Karlsruhe, Germany) was used to characterize the functional groups in the range of 600 to 4000 cm^−1^ from 64 scans in reflection mode. The surface morphology of the separator was studied by Scanning Electron Microscopy (SEM, S4800, Hitachi, Tokyo, Japan) at a working distance of 5 mm and an accelerating voltage of 3 kV, where samples were sputtered with a 10 nm thin gold-palladium layer prior. A contact angle system (OCA20, Dataphysics, Stuttgart, Germany) was used to evaluate the wettability after 1 s of the electrolyte droplet, where 1 M NaClO_4_ in propylene carbonate (PC) and ethylene carbonate (EC) (EC:PC = 1:1, *v*/*v*) was the electrolyte. Mechanical property of the separators was measured using universal tensile tester (AG-Xplus 50KN, Shimadzu, Kyoto, Japan) at a stretching speed of 5 mm/min at room temperature.

The electrolyte uptake rate was obtained by immersing the separator in electrolyte of 1 M NaClO_4_ in EC/PC for 2 h and calculated using the following Equation (1) [22]:

(1) $$\mathrm{Uptake}\left(\%\right)=\frac{m_{1}-m_{0}}{m_{0}}\times 100\%$$

where *m*_0_ represents the original mass of separator, and *m*_1_ is the mass of separator after immersion.

The porosity of the separator was calculated by immersing the separator in n-butanol for 1 h and calculating the weight change using the Equation (2) [13]:

(2) $$\mathrm{Porosity}\left(\%\right)=\frac{m-m_{0}}{\rho_{b}\times V_{m}}\times 100\%$$

where *m* and *m*_0_ represent the mass of the wet separator and the dry separator, respectively, *ρ_b_* stands for the density of n-butanol, and *V_m_* indicates the volume of the separator.

The specific surface area of the samples was studied by the Brunauer–Emett–Teller (BET) method, and the pore size distribution of the samples was calculated by the Barrett–Joyner–Halenda (BJH) method [32].

### 3.5. Electrochemical Measurements

The electrochemical impedance spectroscopy (EIS) was conducted using CHI760E workstation (Shanghai Chenhua Instrument Limited, Shanghai, China) from 0.01 Hz to 1 MHz with an amplitude of 5 mV. A symmetric stainless/separator/stainless (SS/separator/SS) configuration was applied with 90 μL electrolyte (1 M NaClO_4_ in EC/PC). The ionic conductivity (σ) was calculated using Equation (3) [26]:

(3) $$\sigma =\frac{t}{R_{b}\times S}$$

where *R_b_* denotes the resistance, *t* represents the thickness of the separator, and *S* represents the overlap area of the separator and the electrode.

The Na^+^ transference number was assessed by EIS and subjected to ∆V = 10 mV bias to monitor the impedance change until it reached the steady state. The transference numbers based on the Equation (4) [23]:

(4) $$t^{+}=\frac{I_{s}\times \left(\Delta V-R_{o}\times I_{o}\right)}{I_{o}\times \left(\Delta V-R_{s}\times I_{s}\right)}$$

where I_s_, I_o_, R_s_ and R_o_ represent the steady state current, initial current, steady state resistance, the resistance before perturbation, respectively.

The electrochemical performances of separator were tested in coin cells (CR2032) by using a hard carbon electrode as the working electrode and a Na metal sheet as the counter electrode. The hard carbon electrode was prepared by mixing hard carbon power with sodium alginate binder and conductive powder (Super P) at a weight ratio of 90:5:5. The cycling performance and rate compatibility of the cells were performed on a LAND CT3001A (Wuhan, China) battery test system.

## 4. Conclusions

In summary, CNFs were prepared by TEMPO oxidation and assembled into the separator using different approaches to alter their pore structure. The TOCNF-HEF separators exhibit excellent electrolyte uptake and wettability due to their highly porous structure and polar groups. Therefore, remarkable electrochemical performance of Na/hard carbon batteries is obtained using a TOCNF-HEF separator, together with an exceptional ionic conductivity and Na^+^ transference number. Compared to commercial glass fiber separators, the sustainable CNF separators demonstrate a higher mechanical property, and better rate performance and cycling stability in the Na/hard carbon half-cell. This work provides the fundamental relationships between pore structure and electrochemical performance of pure cellulose separators, and also demonstrates the great potential of applying CNF separators in SIBs.

## Figures and Tables

**Table 1:** Physical properties of four TOCNF separators at room temperature.

Parameter	TOCNF-F	TOCNF-E	TOCNF-EF	TOCNF-HEF
Thickness (μm)	80	150	240	250
Porosity (%)	37.1	57.1	70.8	74.6
Uptake (%)	206.2	547.7	776.7	978.8
Contact angle	64.7	17.8	12.5	0

**Table 2:** Comparison with other separators reported in literatures for SIB.

Separator	Ionic Conductivity (mS/cm)	Na^+^ Transference Number	Tensile Strength (MPa)	Electrolyte Uptake (wt%)	Reference
TOCNF-HEF	3.41	0.88	4.49	>800	This work
Glass fiber	6.11	0.80	0.18	>800	This work
Porous Ceramic	8.11	0.80	0.9	175	[26]
Celgard 2730	0.16	0.17	35.3	50	[27]
PVDF	0.74	-	-	341	[28]
CMC/HEC	3.83	-	-	131.4	[12]
ZrO_2_@MCA	2.23	-	1.15	409.2	[13]
Agarose/PVA	1.21	-	10.4	302.7	[22]
Cellulose-PAN-Al_2_O_3_	0.75	0.78	-	286	[29]

## Data Availability

Not applicable.

## Sample Availability

TOCNF suspension and separators are available from the authors.
